# Influence of Different Beverages on the Color Stability of Nanocomposite Denture Base Materials

**DOI:** 10.1155/2021/5861848

**Published:** 2021-11-11

**Authors:** Emad Azmy, Mohamed Reda Zaki Al-kholy, Mohammed M. Gad, Ahmad M. Al-Thobity, Abdel-Naser M. Emam, Mohamed Ahmed Helal

**Affiliations:** ^1^Elmarg Students' Clinic, General Authority of Health Insurance, Western Elmarg Area, Cairo, Egypt; ^2^Department of Removable Prosthodontics, Faculty of Dental Medicine, Al-Azhar University, AlmokhyamAldaem St., Nasr Road, 11884 Nasr City, Cairo, Egypt; ^3^Department of Substitutive Dental Sciences, College of Dentistry, Imam Abdulrahman Bin Faisal University, P.O. Box 1982, Dammam 31441, Saudi Arabia

## Abstract

**Background:**

The effect of beverages on nanocomposite denture base materials is neglected. Therefore, this study aimed to investigate the influence of different beverages (coffee, tea, cola, and mineral water) on the color stability of nanoparticles-modified denture base materials (DBMs).

**Materials and Methods:**

A total of 280 specimens (*n* = 10/group) were prepared from heat-polymerized acrylic resin modified with different concentrations (3% and 7%) of zirconium dioxide (nano-ZrO_2_), titanium dioxide (nano-TiO_2_), and silicon dioxide (nano-SiO_2_) nanoparticles, while 0% was taken as a control. Color change (∆E) of the specimens was evaluated after simulating 6-month immersion time in four commonly used beverages, coffee, tea, cola, and mineral water, as experimental groups. Color stability was measured using a spectrophotometer, and then values were converted to National Bureau of Standards units (NBS units). The one-way ANOVA test was applied to compare color change (ΔE) results followed by Bonferroni's post hoc test (*α* = 0.05).

**Results:**

The results showed that the heat-polymerized acrylic resin modified with different types of nanoparticles showed lower color changes after being immersed in beverage solutions compared to the unmodified group (*P* < 0.001), so the color stability of heat-polymerized acrylic resin was significantly enhanced by the addition of several nanoparticles; nano-ZrO_2_ showed the lowest ΔE followed by nano-TiO_2_ and then nano-SiO_2_. Regardless of the filler type, 3% concentration showed lower mean ΔE than 7% concentration. Regarding the beverage solutions, the greatest color change was found in the coffee group followed by tea and cola, while water showed the least changes.

**Conclusion:**

Modification of heat-polymerized acrylic resin with certain amounts of nano-ZrO_2_, nano-TiO_2_, and nano-SiO_2_ may be useful in improving color stability.

## 1. Introduction

Although polymethyl-methacrylate (PMMA) resin is the material of choice used in fabrication of denture bases, it possesses certain drawbacks such as weak flexural and surface properties, residual monomer, surface porosity, and color instability [1]. Recent trends are directed toward nanoparticles (NPs) incorporation into denture base materials (DBMs) to produce nanocomposites with reasonable properties. It has been found that nanoparticles such as zirconium dioxide nanoparticles (nano-ZrO_2_), titanium dioxide nanoparticles (nano-TiO_2_), and silicon dioxide nanoparticles (nano-SiO_2_) could improve the mechanical and physical properties of nanocomposite PMMA denture base materials [2, 3].

Nano-ZrO_2_is used for acrylic resin reinforcement due to their properties such as high strength, biocompatibility, and esthetic acceptability [3]. Nano-ZrO_2_ is white crystalline metal oxide, polymorphic in nature, and remains without changes in its chemistry at different temperatures [4]. Different studies have demonstrated that 2.5–5% nano-ZrO_2_reinforcement enhanced the mechanical and physical properties of PMMA/ZrO_2_ nanocomposite, and the concentration of nanoparticles was a crucial factor in determining the final properties [5, 6]. Also, it was found that an increase in abrasive wear resistance was observed at 3 and 5wt% nano-ZrO_2_ concentrations, which could be referring to the physical properties of nano-ZrO_2_ [7]. Ihab et al. found that significant color differences between unmodified specimens and nano-ZrO_2_-modified specimens at different immersion solutions and ∆E were increased as nano-ZrO_2_ concentration increased [8].

Nano-TiO_2_ are preferred in dentistry because of their excellent mechanical properties such as corrosion resistant, high microhardness, its white color, light weight, low toxicity, appropriate antimicrobial properties, high stability, and efficiency, as well as availability and low cost [3, 9]. The addition of nano-TiO_2_ to the polymeric material has been shown to affect the electrical, optical, chemical, and physical properties of the hybrid material [9, 10]. It was concluded that the specimens of heat-polymerized PMMA reinforced with different concentrations (1 wt.%, 2 wt.%, and 5 wt.%) of nano-TiO_2_ showed superior flexural strength than those of unreinforced PMMA [11].

Nano-SiO_2_ is one of the most abundant oxide materials in the earth's crust, having good abrasion resistance, electrical insulation, and good thermal stability, so it had been successfully incorporated with PMMA DBMs [12]. The previous studies have reported improving effects on the mechanical (impact, flexural strengths, and surface hardness), optical, and thermal properties of PMMA modified with nano-SiO_2_ [9, 13, 14].

Color stability is considered as one of the most essential clinical merits of all dental materials, and any significant changes in the color are indicative of aging or damaged materials [15]. Color stability is a material status to maintain its color regardless of environmental effect. The color changes of dental polymers may be caused as a result of intrinsic and extrinsic factors: the intrinsic factors as color changes of the dental materials themselves with changing its matrix because of physical and chemical conditions as thermal and humidity alterations that happen during aging; however, the extrinsic factors include the processes as absorption and adsorption of discoloration agents; accordingly, DBMs are required to have adequate color stability to achieve optimal esthetics and serviceability [1, 16].

The different beverages as coffee, tea, wine, and some artificial dyes in food rapidly enhance the staining of DBMs, and this change in color can be considered as an indicator of aging or damage of a material [1, 17]. Renato et al. concluded that the material's composition, staining solution, and immersion time had significant effect on the color stability after immersion for 30 days, where the coffee solution displayed staining ability more than the tea solution [18]. Also, Imirzalioglu et al. concluded that the effect of staining solutions (saliva; control group, saliva + tea, saliva + coffee, saliva + nicotine) on the color of PMMA and soft lining materials was perceivable by the human eye (∆*E* > 1); however, the color shifts of all tested materials were clinically acceptable (∆*E* < 3.7) except for soft liner in nicotine, which was not clinically acceptable over time [19]. In addition, thermocycling in the oral cavity leads to multiple shrinkage and distension of the material that induces material degradation and color alterations [20].

Since limited data are available regarding the beverages' effect on the color stability of nano-ZrO_2_, nano-TiO_2_, and nano-SiO_2_ nanocomposite PMMA. Hence, this *in vitro* study designed to investigate the influence of different beverages; coffee, tea, and Coca-Cola, and mineral water on the color stability of heat-polymerized acrylic resin reinforced with different concentrations of nanofillers (ZrO_2_, TiO_2_, and SiO_2_). The null hypothesis was that beverages effect on the color stability of nanofillers-modified denture base materials would be insignificant.

## 2. Materials and Methods

All materials used in this investigation and their specifications are listed in Table 1. According to previous studies [21, 22], the sample size calculation revealed that a total of 280 specimens were required to conduct the current study, 70 specimens for each immersion solutions. According to the type of nanoparticles, the specimens of the heat-polymerized acrylic resin (Vertex) were divided into four main groups; the 1^st^ group was control group; however, each of the 2^nd^, 3^rd^, and 4^th^ groups was subdivided into two subgroups according to the nanoparticles concentration as described in Table 2.

### 2.1. PMMA Nanocomposite Preparation

Nano-ZrO_2_, nano-TiO_2_, and nano-SiO_2_ were treated separately by using silane coupling agent (3-trimethoxysilyl-propyl-methacrylate (TMSPM)) (Shanghai Richem International Co., Ltd., Shanghai, China) to generate reactive groups on their surfaces to permit better adhesion between NPs and resin matrix. The TMSPM was dissolved in acetone to secure the fact that it would evenly coat the surfaces of the NPs, and then NPs were collected to the TMSPM-acetone solution and stirred with a magnetic stirrer for 60 min. A rotary evaporator (Rotavapor® R-300, Buchi AG, Flawil, Switzerland) was used for eliminating the solvent under vacuum for 30 min at 60°C and 150 rpm. After the sample had dried, it was heated at 120°C for 2 hours and then maintained on the bench at room temperature till the sample was cooled to get the surface-treated NPs [3, 23]. Silanated NPs were weighted using electronic balance (Denver instrument, Göttingen, Germany) of 0.0001 gm accuracy to be added in 3wt% and 7wt% concentrations of acrylic powder. Each NPs and acrylic resin powder were initially mixed together using a mortar and pestle followed by meticulously stirring for 30 min to ensure the homogeneity of the mix and uniformity of color.

### 2.2. Specimen Processing

Metallic dies (20 mm × 2 mm diameter and thickness, respectively) were prepared [24] and used for fabrication of the disc specimens as follows: the metallic dies were painted with separating medium, flasked, and invested in type III dental stone (Kromotypo3, LASCOD, Florence, Italy) and then removed from the flask after setting of the dental stone leaving mould spaces having the same dimensions of the metallic patterns. According to the manufacturer's instructions, the nanocomposite mixture was mixed with the monomer; after that, it was packed at dough stage followed by polymerization for 20 min at 100°C. After complete polymerization and prior to deflasking, the flasks were bench-cooled at room temperature. The specimens were removed from the flasks and cleaned from stone particles. For finishing the specimens, the excess resin was removed using a tungsten carbide bur, followed by wet silicon carbide papers (600-grit, 800-grit, 1000-grit, and 1200-grit). To mimic laboratory procedures, only one surface was wet, polished using a cloth wheel with pumice. All specimens were visually examined; any one with internal or external porosities, warpage, broken edges, altered dimensions, or surface defects was excluded from the study. The accepted specimens were measured again with a digital caliper (Mitutoyo Corp, Tokyo, Japan) with an accuracy of 0.01 mm at 3 different places to verify the correct dimensions; then, the specimens were kept in distilled water at 37°C for 48 h [24].

All specimens were subjected to color measurements before exposure to beverage solutions and considered as baseline using a color reflectance spectrophotometer with computer software (SpectraMagic NX, RM2002QC, Konica Minolta Corp., Ramsey, Japan). According to the manufacturer's instructions, the colorimeter was calibrated before starting any measurement session. All measurements were made with samples resting on a standard white background plate (no. 21633347, Konica Minolta Corp., Ramsey, NJ) with background lights turned on. Each specimen was set in the view port of the spectrophotometer, and measurements of *L*^*∗*^, *a*^*∗*^, and *b*^*∗*^ values of each sample were obtained. The measurement was repeated 3 times, and the mean values of the *L*^*∗*^, *a*^*∗*^, and *b*^*∗*^ data were calculated [25].

**Figure 1:**
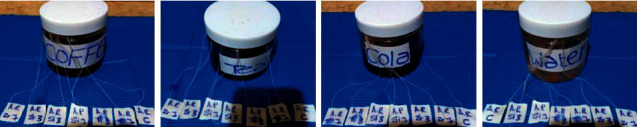
Samples suspended in four different solutions, where AR = heat cured acrylic resin, *C* = control sample (unmodified samples), Zr= ZrO_2_, Ti= TiO_2_, Si= SiO_2_, and 3 and 7 refer to wt.%.

After that, each subgroup was stored for six days as a standard time to simulate consumption of the drink over six months (24 hours' storage time simulated a month of drink consumption) [26]. Four different daily consumed beverages, coffee, tea, Coca-Cola, and mineral water, were prepared in this study as mentioned in Table 3. Each specimen was suspended and immersed in the solutions by means of threads, so the specimen was not in contact with the container or other specimens, and at its end, there is a label indicating that the codes of the specimen were present. All jars were labelled indicating the type of solutions and stored in a 37°C chamber to mimic the oral environment and renewed daily. All solutions were prepared by the same operator for the six days to minimize variances/errors in methodology (Figure 1) [1, 26].

On the day of assessment, the samples were removed from the staining solutions and dried; then, the second color evaluation (*T*_1_) was done as previously explained. The differences in the individual coordinate parameters between baseline (control) and after immersion in coloring solutions (*T*_1_) were calculated (∆*E*) from the following equation: ∆*E* = [(∆*L* (*L*_experiment_ – *L*_base_))^2^ + (∆*a* (*a*_experiment_ – *a*_base_))^2^ + (∆*b* (*b*_experiment_ – *b*_base_))^2^]^1/2^.

Finally, the data were converted to NBS units to relate the color alterations (∆*E*) to the clinical environment as in Table 4 using the following formula [27]:

(1) $$\begin{array}{c} \text{NBS units}=\Delta E^{*}\times 0.92. \end{array}$$

Data were collected and explored for normality by checking its distribution using tests of normality (Kolmogorov–Smirnov and Shapiro–Wilk tests). Data were shown as mean and standard deviation (SD) values. For parametric data, one-way and repeated measures ANOVA tests were employed to compare the groups. Bonferroni's post hoc test was applied for pairwise comparisons when the ANOVA test is significant (*P* ≤ 0.05). Statistical analysis was performed with IBM SPSS Statistics for Windows, Version 23.0. Armonk, NY : IBM Corp.

## 3. Results

**Figure 2:**
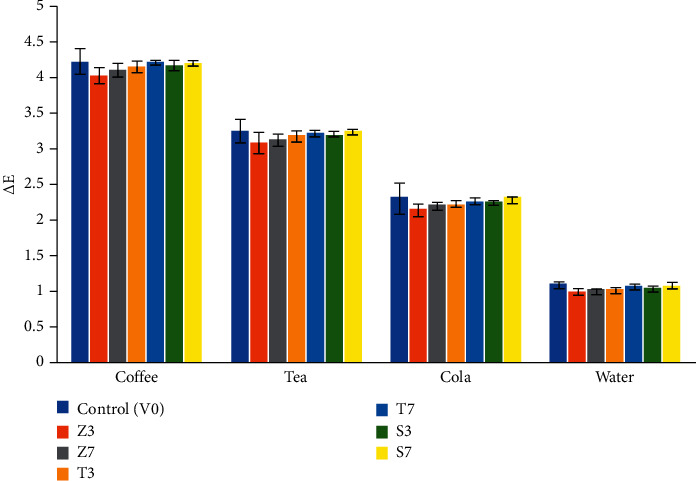
Bar chart representing mean and standard deviation values for color changes (ΔE) in the different acrylic resin groups.

Mean values, SD, and significance of ΔE between all groups and beverages effect are listed in Table 5 and Figure 2. The results of Bonferroni's post hoc test for pairwise comparisons between different groups are listed in Table 6. After immersion in coffee, one-way ANOVA test showed statistically significant difference between ΔE of the different groups (*P* value <0.001, Effect size = 0.326), while the pairwise comparisons between the groups using Bonferroni's post hoc test revealed that there were nonstatistically significant differences between control group (V0) and Z7, T3, T7, S3, and S7 subgroups and a statistically significant difference with Z3. However, there were statistically significant differences between Z3 subgroup with T7, S3, and S7.

After immersion in tea, there was a statistically significant difference between Δ*E* of the different groups (*P* value = 0.002, Effect size = 0.274). The control group showed the highest mean Δ*E* with nonstatistically significant difference from Z7, T3, T7, S3, and S7 subgroups and a statistically significant difference from Z3 subgroup. Z3 subgroup showed the lowest mean Δ*E* with nonstatistically significant difference from Z7, T3, T7, and S3.

After immersion in cola, there was a statistically significant difference between Δ*E* of the different groups (*P* value = 0.008, Effect size = 0.235). The control group showed the highest mean Δ*E* with nonstatistically significant difference from Z7, T3, T7, S3, and S7 subgroups (*P* > 0.05) and a statistically significant difference from Z3 subgroup (*P* < 0.05). Z3 subgroup showed the lowest mean Δ*E* with nonstatistically significant difference from Z7, T3%, T7, and S3.

After immersion in water, there was a statistically significant difference between Δ*E* of the different groups (*P* value <0.001, Effect size = 0.439). The control group showed the highest mean Δ*E* with nonstatistically significant difference from T7, S3, and S7 subgroups and a statistically significant difference from other groups. Z3 subgroup showed the lowest mean ΔE with nonstatistically significant difference from Z7, T3, and S3.

According to NBS findings, regarding coffee and tea groups, NBS values were >3, so marked color changes in these beverages were visually perceptible, which is considered clinically unacceptable. Regarding the cola group, the NBS lies between 1.5 and 3, so noticeable changes were observed, while, in the water group, a slight change was observed.

## 4. Discussion

The esthetic appearance of the prosthesis is a critical factor to meet the patients' expectations, and the color changes of DBMs may result in patient dissatisfaction [15]. Staining mechanism of DBMs has been explained by sorption of liquids and expansion of polymeric matrix and motion of staining agent toward polymeric chains [28]. The DBMs are good environments for colonization of different kinds of microorganisms, so different disinfectants as chlorhexidine are used to control this biofilm, but it was found that the roughness of denture material is increased by using these disinfectants resulting in more stainability of DBMs; furthermore, these substances may affect the color of the dental acrylic as well as the mechanical properties of the PMMA resin [29, 30]. Metal oxides NPs were selected for their best antibacterial activity, unique biological, physical, optical properties, and inertness compared to their macro molecules.

The present study is an attempt to investigate the influence of the coffee, tea, cola, and water immersion on the color stability of heat-polymerized resin DBM reinforced with different concentrations of nano-ZrO_2_, nano-TiO_2_, and nano-SiO_2_ carried out for a 6-month simulation. Coffee, tea, cola, and mineral water were selected as beverage solutions in this study, because they are daily used and were usually used in the *in vitro* studies.

Evaluation of color alterations can be measured visually or by using certain instrumentation. Colorimeters and spectrophotometers are commonly used to evaluate color changes of dental materials, as it eliminates subjective interpretations and allows identification of minor color alterations [31]. The Commission Internationale de l'Eclairage (CIE) L^*∗*^, a^*∗*^, b^*∗*^ is a constant color scale that comprises all the colors visible to the human eye. Thus, it is a suitable tool to assess color changes in dental materials [19, 32].

After immersion of the specimens in coffee, tea, cola, and mineral water, the results of this study revealed that there were significant differences between the Δ*E* of different groups (*P* ≤ 0.05). Hence, the null hypothesis of the present study was rejected, where the color stability of heat cured PMMA DBMs was significantly enhanced by nanofillers incorporations when immersed in different beverages.

**Figure 3:**
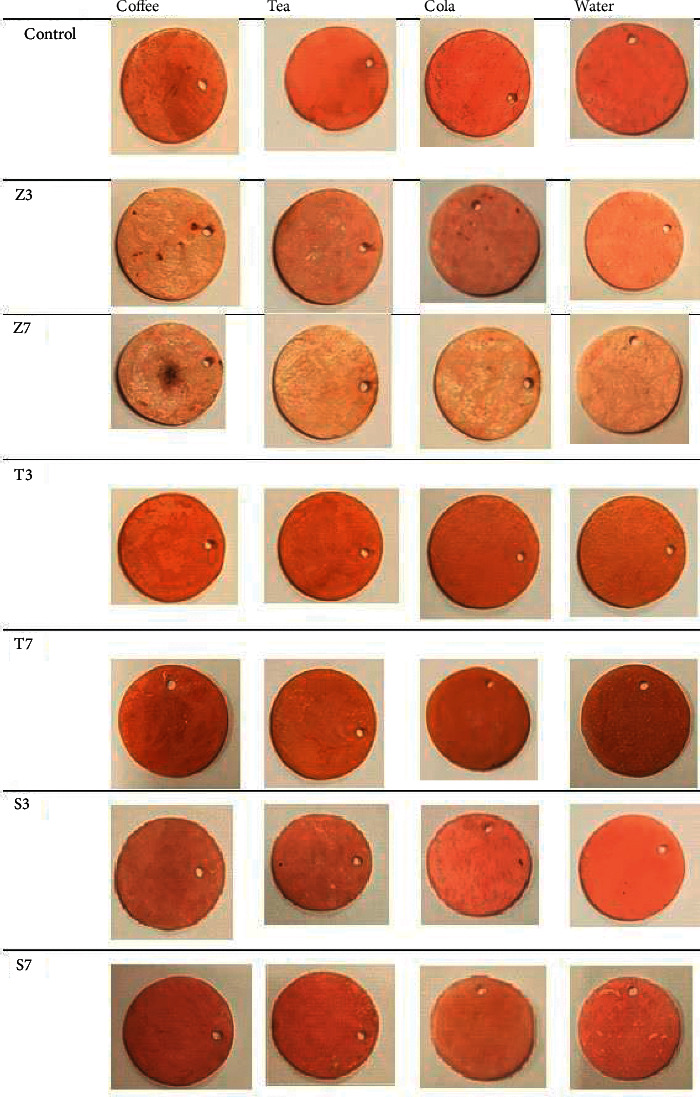
Color change after immersion in different beverages.

According the results of the current study, the specimens immersed in coffee showed great color changes followed by tea and cola; this may be explained, as discoloration by coffee occurred as a result of both surface absorption and adsorption of colorants and fine coffee particles deposits into pits of PMMA resin DBM (Figure 3). The less polar colorants and water-soluble polyphenols as tannin, caffeine, and caffeinic acid of the coffee are more compatible with polymer matrices, so they might have penetrated the material deeply [26]. This result was in agreement with many previous studies [33–35], which demonstrated that the coffee solution produced more discoloration for the specimens of heat-polymerized resin DBM more than tea solution.

Recently, Ayaz et al. reported that heat and microwave polymerized PMMA can show different color stability, and immersion in coffee and denture cleaner solutions can cause noticeable color changes [36]. In the present study, coffee and tea were not containing sugar; a previous study has showed that sugar may create further staining most probably by promoting the adhesion of the colorant agents to the surface [26]. However, in another study, coffee with cream and sugar exhibited the least discoloration especially in the aged after 7 days of storage; this could be due to the whitening feature of the creamer [17]. Zuo et al. reported that all specimens of heat-polymerized resins DBM were stained or discolored to varying degrees after immersion in tea, red wine, coffee, and cola, and the discoloration was noticed to be a time-dependent condition and increased with extended immersion times (28 days) [37].

In the current study, there was a significant effect for the tea on the color stability of the specimens of the different groups; this was in agreement with Altinci et al., who reported that the black tea led to a noticeable discoloration, and the change was also noticeable for the aged specimens. In addition, sour cherry juice and cola produced a similar color change for the aged after 7 days of storage [17]. On the other hand, our finding was in disagreement with previous studies [21, 38] reporting that the tea solution has more staining capacity on the resin DBM than the coffee solution, which may be due to higher polarity components of tea more than that of coffee. This may be due to the differences in the methodology, concentrations beverages, or different type of DBM used. The present study showed discoloration effect of the cola on the specimens of different groups but less than the coffee or tea. This was in agreement with Keyfet al., who reported that the extremely low pH of Pepsi can lead to discoloration of the materials, but less change than in coffee and tea solutions. The reason for less staining effect of cola than other beverages may be due to the removal of accumulated layers that tend to break away from the surface of samples and return to the beverage solutions [38].

In the present study, the least color change was noticed in specimens immersed in mineral water; this may be due to the fact that there are no colorant substances that may participate on the specimen surface leading to its discoloration; also the pH of the water may cause a minimum roughness on the surface due to neutrality [16, 22]. This finding was in accord with the study of Keyf and Etikan [38]. The slight color change in mineral water with time may occur due to presence of water, which tends to soften the polymer by causing swelling of the network and loosening the frictional forces between polymer chain [39].

Based on our results, color changes were reduced by the addition of nanoparticles; nano-ZrO_2_ showed the lowest Δ*E* followed by nano-TiO_2_ then nano-SiO_2_. Regardless of the filler type, 3% concentration showed lower mean Δ*E* than 7% concentration. It was found that the presence of inorganic fillers incorporated within the material leads to an increase in the density, and porosity was decreased, so less stain absorption of this adverse relationship between density and porosity was approved by Keller and Lautenschlag [40]. Furthermore, Hamid and Abdul Rahman reported that, with the addition of nano-ZrO_2_, there was a decreased apparent porosity leading to less stain absorption [41].

Nano-TiO_2_ particles can increase the color stability and esthetic of acrylic DBM through preventing the passage of ultraviolet light, reducing the color degradation of the pigments inside the polymer and preventing the discoloration [42]. Laura et al. added nano-TiO_2_, for improving the properties of PMMA DBM; the result showed a strong reduction of porosity with the introduction of nanosized metal oxide pigments [43]. Rio et al. studied the effect of different solution artificial saliva, coffee, cola, and alcohol on the PMMA DBM and found that TiO_2_ coating slows down the process of color change of heat-polymerized acrylic resin, thus increasing the life of the prosthesis, which comes in agreement with our findings [44]. Furthermore, it was found that nano-TiO_2_ and nano-SiO_2_ can improve the optical and physical properties of the polymeric materials [2]. Alexandros et al. reported that reinforcement of the selected PMMA resin for interim restorations with SiO_2_ nanoparticles resulted in significantly lower color change [45].

From the clinical view, modification of PMMA DBM with nano-ZrO_2_, nano-TiO_2_, or nano-SiO_2_ may decrease its stainability, which is benefitted in the increase of the life span of removable dentures, as well as patient acceptance. Beverages (coffee, tea, cola, and mineral water) significantly increase stainability of DBM, which may be considered as an indicator of aging or damage of a material, so minimizing drinking of such beverages may be advantageous for denture wearers for long-term color stability. With advanced digital technology, this nanocomposite could be recommended following digitally planned and milled implant-prosthesis or total prosthesis, which could be applied for provisional and definitive treatment [46].

The limitations of this study include the lack of human saliva and denture biofilm that may affect the color change results leading to inaccurate prediction of the clinical performance of the materials being tested, so the presented findings are only a promising starting point for further investigations. Also, we use a simple disc shaped specimen, which does not reflect the shape of an actual. Future work should involve the polymerization technique and other types of denture base and nanoparticles. In addition, the polishing effect should be investigated.

## 5. Conclusion

According to the limitations of this study, the following conclusions could be drawn:

- Modification of heat-polymerized acrylic resin with 3% of nano-ZrO_2_, 3% nano-TiO_2_, and 7% of nano-ZrO_2_ may be useful in improving its color stability when compared with conventional acrylic resin.
- The coffee solution has more chromatic effect on the specimens of heat-polymerized resin DBM greater than that of tea and cola.
- The mineral water has the least effect on the color changing of the specimens of heat-polymerized resin DBM.

## Figures and Tables

**Table 1:** The manufacturer's specifications of metal oxide nanoparticles.

Material	Zirconium oxide (ZrO_2_)	Titanium oxide (TiO_2_)	Silica oxide (SiO_2_)
Color	White	White	White
Form	Powder	Powder	Powder
Average size (TEM)	12 ± 3 nm	15 ± 3 nm	21 ± 3 nm
Purity	Purity >99%	Purity >99%	Purity >99%
Shape (TEM)	Spherical	Spherical	Spherical
Crystal system	Tetragonal	Anatase 95–97%, brookite 5–3%	Amorphous
Manufacturer	NanoGATE, Egypt		

**Table 2:** Grouping and coding of different variables.

Variables solution	1^st^ group (control)	2^nd^ group		3^rd^ group		4^th^ group		Total
		ZrO_2_ (Z)		TiO_2_ (T)		SiO_2_ (S)		
	V0	3%	7%	3%	7%	3%	7%	
		Z3	Z7	T3	T7	S3	S7	
Coffee	10 samples	10	10	10	10	10	10	70
Tea	10	10	10	10	10	10	10	70
Coca-Cola	10	10	10	10	10	10	10	70
Mineral water	10	10	10	10	10	10	10	70
Total	40	40	40	40	40	40	40	280

**Table 3:** Beverage preparation protocol.

Beverage	Brand name	Preparation	Immersion time	Temperature (°C)
Coffee	Abu Auf (Sama Trading Co., Egypt)	2 g of coffee was dissolved in 200 ml of distilled boiling water for 2 minutes then filtered to remove the dust	Six days	37
Tea	Lipton (yellow label tea, Lipton Co., Egypt)	2 g of tea was dissolved in 200 ml of distilled boiling water for 2 minutes then filtered to remove the dust		
Cola	Pepsi (Coca-Cola Co., Egypt)	Ready-made		
Water	Dasani Coca-Cola Bottling Co., Egypt	Ready-made		

**Table 4:** NBS rating system for expressing the color system.

NBS units	Remarks on color difference
0.0–0.5	Trace change
0.5–1.5	Slight change
1.5–3	Noticeable change
3–6	Marked change
6–12	Extremely marked change
12 or more	Change to other colors

**Table 5:** Descriptive statistics and results of the one-way ANOVA test for comparison between color changes (ΔE) in the different acrylic resin groups after immersion in different media and relations between ΔE and NBS for all groups.

Group	Coffee		Tea		Cola		Water	
	Mean ± SD	NBS	Mean ± SD	NBS	Mean ± SD	NBS	Mean ± SD	NBS
Control (V0)	4.22 ± 0.18^A^	3.9	3.25 ± 0.16^A^	3	2.30 ± 0.22^A^	2.1	1.09 ± 0.04^A^	1
Z3	4.02 ± 0.11^B^	3.7	3.08 ± 0.15^B^	2.8	2.14 ± 0.08^B^	2	0.99 ± 0.05^C^	0.9
Z7	4.10 ± 0.10^AB^	3.8	3.12 ± 0.08^AB^	2.9	2.19 ± 0.06^AB^	2	1.00 ± 0.04^C^	0.9
T3	4.14 ± 0.08^AB^	3.8	3.18 ± 0.08^AB^	2.9	2.22 ± 0.05^AB^	2	1.01 ± 0.04^BC^	0.9
T7	4.20 ± 0.03^A^	3.9	3.21 ± 0.05^AB^	3	2.26 ± 0.05^AB^	2.1	1.06 ± 0.04^AB^	0.97
S3	4.16 ± 0.07^A^	3.8	3.20 ± 0.04^AB^	2.9	2.24 ± 0.03^AB^	2.1	1.04 ± 0.04^ABC^	0.95
S7	4.20 ± 0.04^A^	3.9	3.23 ± 0.04^A^	3	2.28 ± 0.04^A^	2.1	1.08 ± 0.05^A^	1
*P* value	<0.001^*∗*^		0.002^*∗*^		0.008^*∗*^		<0.001^*∗*^	
Effect size (Eta squared)	0.326		0.274		0.235		0.439	

^*∗*^Significant at *P* ≤ 0.05. Different superscripts in the same column indicate statistically significant difference between groups.

**Table 6:** Results of Bonferroni's post hoc test for pairwise comparisons between different groups.

Group	Coffee	Tea	Cola	Water
	*P* value	*P* value	*P* value	*P* value
V0 vs. Z3	<0.001^*∗*^	0.004^*∗*^	0.008^*∗*^	<0.001^*∗*^
V0 vs. Z7	0.119	0.056	0.158	<0.001^*∗*^
V0 vs. T3	0.559	0.673	0.516	0.002^*∗*^
V0 vs. T7	0.999	0.967	0.966	0.725
V0 vs. S3	0.829	0.908	0.804	0.159
V0 vs. S7	0.998	0.999	0.999	0.999
Z3 vs. Z7	0.486	0.967	0.914	0.999
Z3 vs. T3	0.092	0.257	0.531	0.947
Z3 vs. T7	0.002^*∗*^	0.056	0.100	0.012^*∗*^
Z3 vs. S3	0.029^*∗*^	0.098	0.261	0.159
Z3 vs. S7	0.003^*∗*^	0.016^*∗*^	0.031^*∗*^	<0.001^*∗*^
Z7 vs. T3	0.972	0.808	0.992	0.999
Z7 vs. T7	0.290	0.379	0.667	0.048^*∗*^
Z7 vs. S3	0.829	0.523	0.906	0.400
Z7 vs. S7	0.338	0.164	0.372	0.002^*∗*^
T3 vs. T7	0.829	0.993	0.966	0.159
T3 vs. S3	0.999	0.999	0.999	0.725
T3 vs. S7	0.870	0.908	0.804	0.012^*∗*^
T7 vs. S3	0.972	1.000	0.999	0.947
T7 vs. S7	1.000	0.999	0.999	0.947
S3 vs. S7	0.984	0.993	0.966	0.400

^*∗*^Significant at *P* ≤ 0.05.

## Data Availability

The data are available from the corresponding author upon reasonable request.
